# Cross‐Modal Transfer as a Window Into the Investigation of Recent Stimulus and Response History in Perceptual Decision‐Making

**DOI:** 10.1111/ejn.70436

**Published:** 2026-02-19

**Authors:** Daniel C. Fitze, Daniel Schlunegger, Fred W. Mast

**Affiliations:** ^1^ Department of Psychology University of Bern Bern Switzerland

**Keywords:** choice history, heading task, perceptual decision‐making, response bias, sequential choice effects, serial dependence

## Abstract

Perceptual decisions are shaped by recent stimulus and response history, yet these history effects vary with experimental design and task structure. Their origins, the processing stages involved, the factors that determine their magnitude and the sources of individual differences require further investigation. We propose a multisensory approach that leverages cross‐modal transfer of history effects as a diagnostic tool to address these open questions. Visual–vestibular stimuli are particularly suitable because both modalities contribute to estimating the body's position in space and they likely share central processing stages. This approach allows for manipulation of response format, independently of sensory stimulation. By moving beyond isolated sensory channels and examine perceptual decisions as they occur in everyday multisensory environments, we provide a framework to investigate when, how and why recent history shapes perception.

## Graphical abstract

Response and stimulus history shape sensory processing, perception and perceptual decision‐making, yet the underlying mechanisms need more research. We propose a visual–vestibular heading discrimination task, allowing for an independent manipulation of response format, sensory input and post‐perceptual processing. Through this conceptual exploration of recent history effects on perceptual decisions, we seek to advance their investigation towards the multimodal interplay of everyday perception.

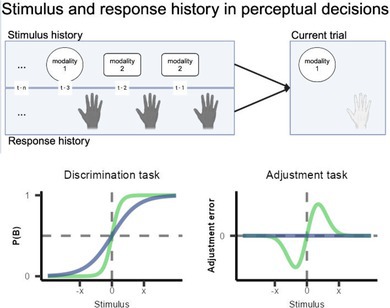

## 1 Introduction

Perception depends not only on the current stimulus but also on recent experience. Substantial evidence demonstrates history effects resulting from a combination of mechanisms, including both attractive and repulsive components with contributions from multiple processing stages (see reviews; Manassi et al. 2023; Pascucci et al. 2023; Wang‐Juan et al. 2022). However, the origins of history effects, the processing stages involved and the sources of individual differences (Guan and Goettker 2024; Kondo et al. 2022; Zhang and Alais 2020) remain actively debated. Numerous studies support perception‐based history effects (e.g., Cicchini et al. 2017, 2021; Collins 2019, 2020, 2022; Fornaciai et al. 2023; Fornaciai and Park 2018; Manassi et al. 2023; Manassi and Whitney 2022; St. John‐Saaltink et al. 2016; Turbett et al. 2019), some of which are not explainable by higher cognitive factors. Conversely, other work has demonstrated the involvement of higher‐level processes (e.g., Akaishi et al. 2014; Braun et al. 2018; Fründ et al. 2014; Schlunegger and Mast 2023; Urai et al. 2019; Urai and Donner 2022). However, isolating the contributions of memory, attention and awareness remains challenging due to methodological confounds and conceptual ambiguities, as the field lacks consensus on the operational definitions of these constructs (Manassi et al. 2023). These findings are based on various tasks, experimental paradigms and stimuli, where subtle changes can substantially alter outcomes. Moreover, research on statistical dependencies between consecutive trials has focused primarily on sensory modalities in isolation. For example, Cicchini et al. (2021) exploited the tilt surround illusion to demonstrate that history effects are formed at higher‐level processes and then propagate down to, and interact with, early sensory processing (for further examples, see Collins 2022; Fornaciai and Park 2018; Murai and Whitney 2021).

Examining cross‐modal transfer offers to approach these questions more directly. Transfer between modalities inherently isolates higher‐level shared processes from modality‐specific sensory mechanisms. This is particularly important because perception is fundamentally multisensory, yet most studies examine within‐modality perception. Unimodal studies typically present numerous repetitions of similar stimuli, which differ along one dimension (e.g., orientation). This entails a high predictability and strong anticipatory processing, which may affect the history effects at study. Moreover, history effects demonstrated across different tasks and stimuli suggest the involvement of shared higher‐level processes—a hypothesis that cross‐modal transfer research can test directly.

Previous research using unimodal paradigms has demonstrated transfer across various visual stimuli (Ceylan et al. 2021) and different objects (Tanrikulu et al. 2023) and between static orientation and motion direction stimuli (You et al. 2023). Interestingly, these within‐modality transfer effects can extend beyond the visual domain (Snyder et al. 2015), with some evidence suggesting overlap across modalities (Bertelson et al. 2003; Vroomen et al. 2007). The available research on cross‐modal transfer reveals a complex picture. Li et al. (2023) demonstrated that sensory and decision‐related transfer occurred only when both preceding and current stimuli belonged to the same modality. Similarly, Fornaciai and Park (2019) observed modality‐specific history effects in a visual numerosity task. Participants compared the number of dots between reference and probe stimuli, with either auditory or visual inducers presented at trial onset. Only visual inducers produced positive history effects, indicating no cross‐modal transfer. However, more recent findings are in support of bidirectional cross‐modal transfer. Hashimoto and Makioka (2025) used a standardized temporal presentation format across modalities, and they found bidirectional transfer between audition and vision. They argued that differences in the stimulus presentation format (temporal vs. spatial) can prevent the occurrence of cross‐modal transfer. Moreover, Lau and Maus (2019) found evidence for partial cross‐modal transfer. Visual and auditory stimuli influenced subsequent responses in visual but not auditory trials. Critically, this auditory‐to‐visual transfer occurred only when the reporting modality was revealed after stimulus presentation, making sensory content from both modalities potentially relevant. Arrighi et al. (2014) demonstrated significant cross‐modal adaptation for numerosity, where visual adaptation transferred to auditory perception and vice versa. Additionally, Shalom‐Sperber et al. (2022) observed negative cross‐modal adaptation (visual–vestibular) with biases away from preceding stimuli, consistent with repulsive stimulus history effects. Taken together, the evidence for cross‐modal transfer is not conclusive, and more research is needed to better understand history effects in the context of cross‐modal dynamics. Transfer indicates shared mechanisms; lack of transfer reveals modality‐specific processes. This precision is important because similar tasks can engage different mechanisms, as shown by spatial versus temporal numerosity judgements (Hashimoto and Makioka 2025). Cross‐modal paradigms thus provide a direct test of whether history effects arise from sensory or decision‐level processes. Importantly, cross‐modal research allows investigation beyond specific unimodal stimuli or tasks to thoroughly characterize the principles determining how recent history shapes perceptual decision‐making.

## 2 Conceptual Approach

The contradictory findings on cross‐modal transfer may partly reflect the use of arbitrarily matched and distal sensory modalities. To address this, we propose passive self‐motion perception as an ideal framework, because it is inherently cross‐modal and the visual and vestibular systems are tightly connected (Angelaki and Cullen 2008). Both senses simultaneously encode self‐motion. The acceleration of the head resulting from self‐motion stimulation is detected by the vestibular system, whereas the corresponding change in the visual field (optical flow) is registered by the visual system. These complementary signals are continuously integrated during natural behaviour (Fetsch et al. 2010; Keshavarzi et al. 2023). Visual–vestibular interaction thus offers critical advantages over other modality pairings for investigating cross‐modal dynamics. The brain requires a unified representation of body position in space, to which both visual and vestibular systems contribute. Computationally speaking, evidence about body position accumulates in a common variable at a higher level. Indeed, integrating visual and vestibular sensory information yields more precise estimates than either modality alone (e.g., Drugowitsch et al. 2014; Gu et al. 2008). Because these sensory systems are intrinsically linked, they are more likely to share central processing stages, providing a more direct window into cross‐modal dynamics. In contrast, arbitrarily paired or distally related modalities require learned associations (e.g., linking high‐pitched tones to objects in the upper visual field). Such parings may maintain separate processing streams until late decision stages, and this can make history effects unlikely to occur. Moreover, learned mappings introduce additional cognitive demands such as associative memory or attention to arbitrary cue–target mappings. The proximity of modalities is also important when examining individual differences. Our focus is on process‐relevant individual differences—those that are directly tied to the mechanisms underlying history effects. Using sensory modalities with high proximity minimizes non‐process‐related variability stemming from additional cognitive processes.

Specifically, in a visual–vestibular heading discrimination paradigm, history effects have been established in each modality independently. Gonzalez et al. (2023) demonstrated that vestibular self‐motion perception exhibits negative history effects, with responses biased away from previous responses (repulsive history effect). Also, visual heading perception shows history effects, with responses biased towards previously perceived headings (Sun et al. 2020; Xu et al. 2022). Shalom‐Sperber et al. (2022) provided first evidence of cross‐modal transfer in heading tasks. However, several key questions remain unsolved: the origins of history effects in the unimodal and multimodal contexts, the processing stages at which cross‐modal transfer occurs, the factors governing when and how strongly history effects arise and transfer and the sources of individual differences. Nevertheless, the visual–vestibular heading discrimination task provides an ideal framework to systematically investigate cross‐modal transfer. Its numerous advantages enable us to (1) investigate stimulus and response history effects within the visual and vestibular modalities, (2) investigate within‐modality transfer of history effects, (3) directly compare the history effects across modalities, (4) combine modalities to investigate the cross‐modal transfer of history effects and (5) implement both a forced‐choice paradigm (direction discrimination) and an adjustment paradigm (position reproduction). Notably, all of this is possible without changing the sensory stimulation. In contrast, most cross‐modal paradigms require fundamentally different stimulus types for different modalities (e.g., oriented gratings for vision and pure tones for audition) or different response paradigms. The heading task is not only a valuable paradigm for examining history effects, but it also points out the importance of the vestibular system, which is sometimes overlooked in the context of multisensory integration.

**FIGURE 1:**
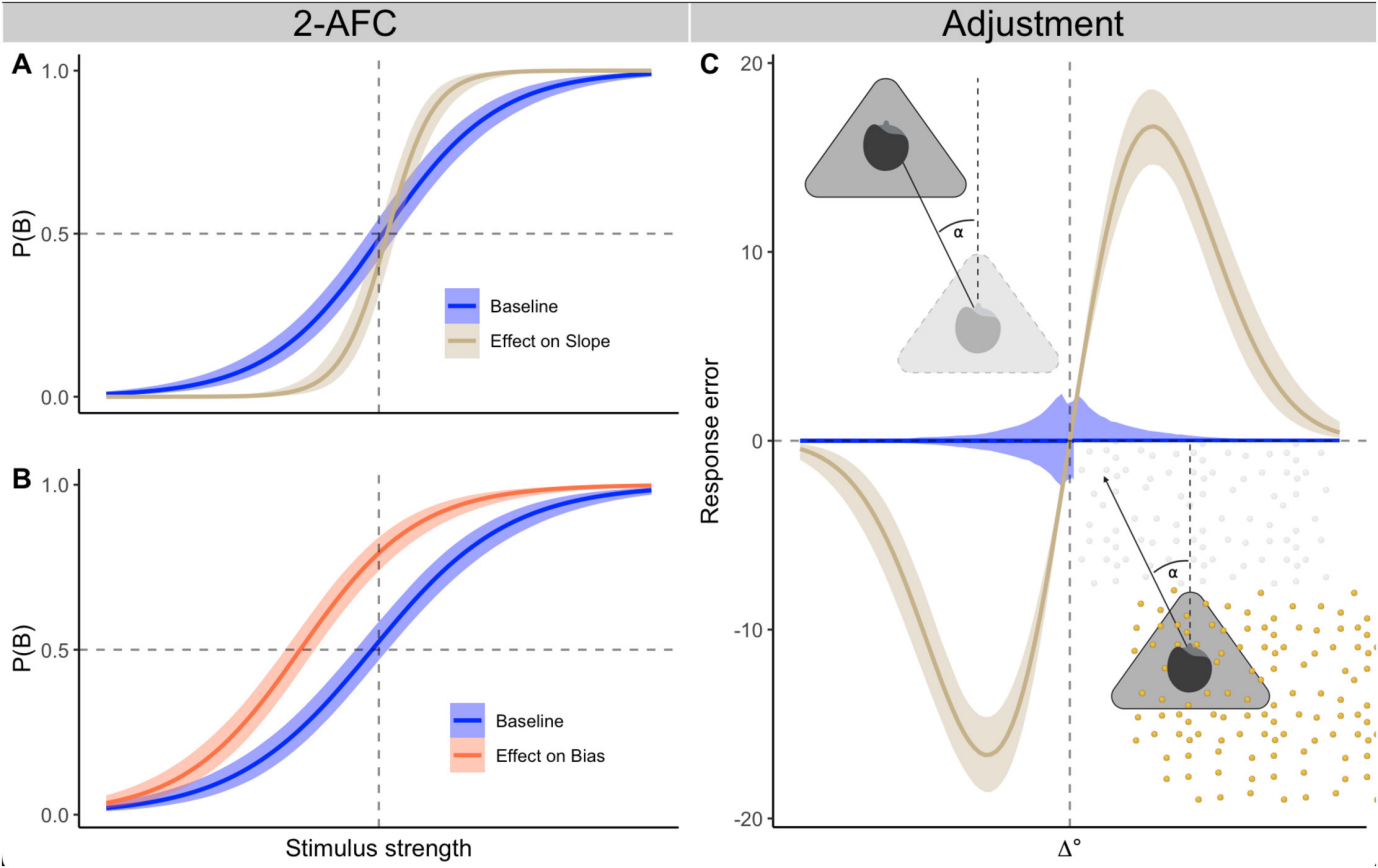
Predicted results. Panels A and B show the influence of recent history in the 2AFC paradigm. Either the slope (A) or the bias (B) of the psychometric function is influenced by history effects. Panel C shows the influence of recent history in the adjustment paradigm, where the amplitude of the reproduction error is increased by history effects. Panel C contains visualizations of vestibular (top) and visual (bottom) heading stimuli. These illustrations were created in BioRender: https://BioRender.com/m27z420.

A typical heading stimulus consists of forward translation with a small lateral component known as the heading angle. Whether encoded visually (optical flow), vestibularly (head acceleration) or both, the passive self‐motion perception is the same. In a heading experiment, repetitions of unimodal and combined stimuli are presented in random order, and the heading angle is varied across trials. All aspects mentioned above (1–5) can be addressed without altering the self‐motion stimulation set‐up. In the direction discrimination paradigm (2AFC), the participants classify the motion direction relative to straight ahead in each trial. Psychometric curves are fit to participants' binary responses (e.g., Li et al. 2023; Schlunegger and Mast 2023). These curves can be conditioned on several variables such as the preceding modality, the identity of the preceding stimulus or the response given in the preceding trial. History effects manifest either in the bias or in the slope parameter of the psychometric curves. Alternatively, instead of judging the motion direction, the participant can reproduce the motion stimulus (heading angle) by using the adjustment paradigm. The heading angle can either be reproduced by moving the visual scene (visual condition) or by moving the motion platform in darkness (vestibular condition). With the adjustment paradigm, the reproduction error (difference between the real motion and its reproduction) is fit with the first derivative of a Gaussian function (Figure 1, Panel C; Fischer and Whitney 2014). The amplitude parameter of this model estimates the magnitude of history effects by relating the adjustment error to the difference in heading angle between the previous and the current stimulus. This model, too, can be conditioned on the variables described above.

What kind of results can be expected? Within the visual and vestibular modalities, (1) we expect a replication of a positive effect towards recent responses combined with repulsion from previous stimuli. As shown in Figure 1, history effects either impact the bias (Panel B; Alais et al. 2017; Taubert et al. 2016) or the slope parameter (Panel B; Fischer and Whitney 2014—Experiment 3) in the discrimination paradigm while increasing the amplitude in the adjustment paradigm (Panel C). Additionally, (2) within‐modality transfer (such as across different motion directions) needs to be replicated. When (3) comparing between the visual and vestibular modality, we expect similar history effects. (4) Cross‐modal transfer will shed light on the origin of history effects. Without cross‐modal transfer, the sensitivity and amplitude will be increased with respect to the baseline. Finally, (5) explicit comparison between forced‐choice and adjustment paradigms will provide insights whether history effects draw on the same underlying mechanisms. We expect that stronger engagement of post‐perceptual processes will lead to stronger history effects. To test this, post‐perceptual processing time must be explicitly manipulated within each paradigm. We propose to achieve this by varying the response delay, as Bliss et al. (2017) demonstrated that longer delays between stimulus and response lead to increased history effects. Additionally, modality specific alterations to the heading stimuli allow further identification of the propagated information's origin. By systematically altering the stimuli in one modality at a time, it can be determined which sensory channel drives the transferred history effect, thereby localizing its origin. Possible alterations are the reliability of the sensory information, which has been manipulated in heading tasks (e.g., Fetsch et al. 2009) and in the context of history effects (Ceylan et al. 2021; Cicchini et al. 2018; Gallagher and Benton 2022; Little and Clifford 2025) or stimulus timing (e.g., Rodriguez and Crane 2021).

## 3 Discussion

Systematic investigation of history effects requires careful experimental control. We propose that multisensory stimuli offer unique theoretical advantages over unimodal approaches. Combining two sensory modalities separates processing stages and enables direct comparison between discrimination and adjustment paradigms using identical multisensory stimulation. Visual–vestibular heading stimuli are particularly well suited for this approach. These modalities naturally converge onto a unified representation of body position in space, and the perception of body position inherently integrates both visual and vestibular signals. This natural integration has three critical implications. First, it ensures that both modalities access shared central processing stages, providing a direct window into cross‐modal dynamics without the confounds introduced by arbitrary learned associations. Second, it enables identical multisensory stimulation across discrimination and adjustment paradigms, allowing direct comparison. Third, the principles of visual–vestibular integration are well characterized (Drugowitsch et al. 2014; Gu et al. 2008), and heading tasks are well established in basic human research (Fetsch et al. 2009) and primate studies (Hou et al. 2019) and in clinical settings (Beylergil et al. 2021). This extensive foundation facilitates the integration of future findings on history effects into broader frameworks of multisensory perception.

Future research needs to systematically map the parameters that govern cross‐modal transfer dynamics. We propose starting with closely related modality pairings, such as visual and vestibular self‐motion perception, and gradually extending to more distant combinations. Proximity between modalities can be operationalized through tight interaction patterns (e.g., audio‐visual speech perception in the McGurk effect). The visual–vestibular heading approach presented in this paper will help to clarify when history effects reflect integrated decision processes versus modality‐specific effects, ultimately enabling prediction of their occurrence, quality and individual variability across different sensory contexts.

The systematic approach illustrated by the heading task will make it possible to predict when history effects occur, how they transfer across modalities and how they are influenced by individual differences. Ultimately, such understanding will support a comprehensive account of how the brain perceives in the rich multisensory environments of everyday life.

## Author Contributions

**Daniel C. Fitze:** conceptualization, methodology, project administration, resources, software, visualization, writing – review and editing. **Daniel Schlunegger:** conceptualization, methodology, project administration, resources, software, visualization, writing – original draft, writing – review and editing. **Fred W. Mast:** conceptualization, funding acquisition, methodology, project administration, resources, software, supervision, visualization, writing – original draft, writing – review and editing.

## Conflicts of Interest

The authors declare no conflicts of interest.

## Data Availability

The discussions and analyses presented are based on previously published studies and theoretical frameworks. References are provided in the manuscript. Code for Figure 1, the graphical abstract and data simulation/analysis can be found here: https://github.com/dafitze/cross‐modal. Part of the graphical abstract and Figure 1 were created in BioRender: https://BioRender.com/m27z420.
